# Systematic optimization of human pluripotent stem cells media using Design of Experiments

**DOI:** 10.1038/srep09834

**Published:** 2015-05-05

**Authors:** Paulo A. Marinho, Thanathom Chailangkarn, Alysson R. Muotri

**Affiliations:** 1 University of California San Diego, School of Medicine, Department of Pediatrics/Rady Children’s Hospital San Diego, Department of Cellular & Molecular Medicine, Stem Cell Program, La Jolla, CA 92093, MC 0695, USA

## Abstract

Human pluripotent stem cells (hPSC) are used to study the early stages of human development *in vitro* and, increasingly due to somatic cell reprogramming, cellular and molecular mechanisms of disease. Cell culture medium is a critical factor for hPSC to maintain pluripotency and self-renewal. Numerous defined culture media have been empirically developed but never systematically optimized for culturing hPSC. We applied design of experiments (DOE), a powerful statistical tool, to improve the medium formulation for hPSC. Using pluripotency and cell growth as read-outs, we determined the optimal concentration of both basic fibroblast growth factor (bFGF) and neuregulin−1 beta 1 (NRG1β1). The resulting formulation, named iDEAL, improved the maintenance and passage of hPSC in both normal and stressful conditions, and affected trimethylated histone 3 lysine 27 (H3K27me3) epigenetic status after genetic reprogramming. It also enhances efficient hPSC plating as single cells. Altogether, iDEAL potentially allows scalable and controllable hPSC culture routine in translational research. Our DOE strategy could also be applied to hPSC differentiation protocols, which often require numerous and complex cell culture media.

Despite the numerous and rapid advances in hPSC technology over the past decade^1^,^2^,^3^,^4^,^5^, culture conditions still rely on empirically formulated media. As an example, the most widely used commercially available feeder free culture medium for hPSCs, mTeSR1, has raised concerns about the accumulation of spontaneous differentiation in the culture, requiring labor-intensive cleaning procedures and unavoidably daily routine of media change^6^ with substantially high cost for culture maintenance. As a consequence, the hPSC field continues to use suboptimal culture conditions that could lead to experimental variation or even mask important observations. One well-known example is the inconsistency of X-chromosome inactivation status in hPSC from different labs. Problems associated with empirically formulated media could be explained by the lack of well-designed optimization steps while evaluating the interactions between manifold components.

DOE is a mathematical technique that can be used to determine the optimal set of conditions across many different changeable parameters^7^,^8^. One of the greatest advantages of the DOE approach is the capacity to reduce the number of experiments needed to identify an optimal set of conditions. For this reason, DOE is routinely used in several fields of study; engineers use DOE to optimize physical structure design^9^,^10^,^11^ and medicinal chemists use DOE to optimize drug formulation^12^,^13^. However, DOE has never been used to optimize hPSC culture conditions. In this work, we sought to improve hPSC culture conditions by optimizing the levels of two well-established growth factors that regulate pluripotency: basic fibroblast growth factor (bFGF)^14^,^15^ and neuregulin-1beta 1 (NRG1β1)^15^.

## Results

### Development of media formulation

**Figure 1:**
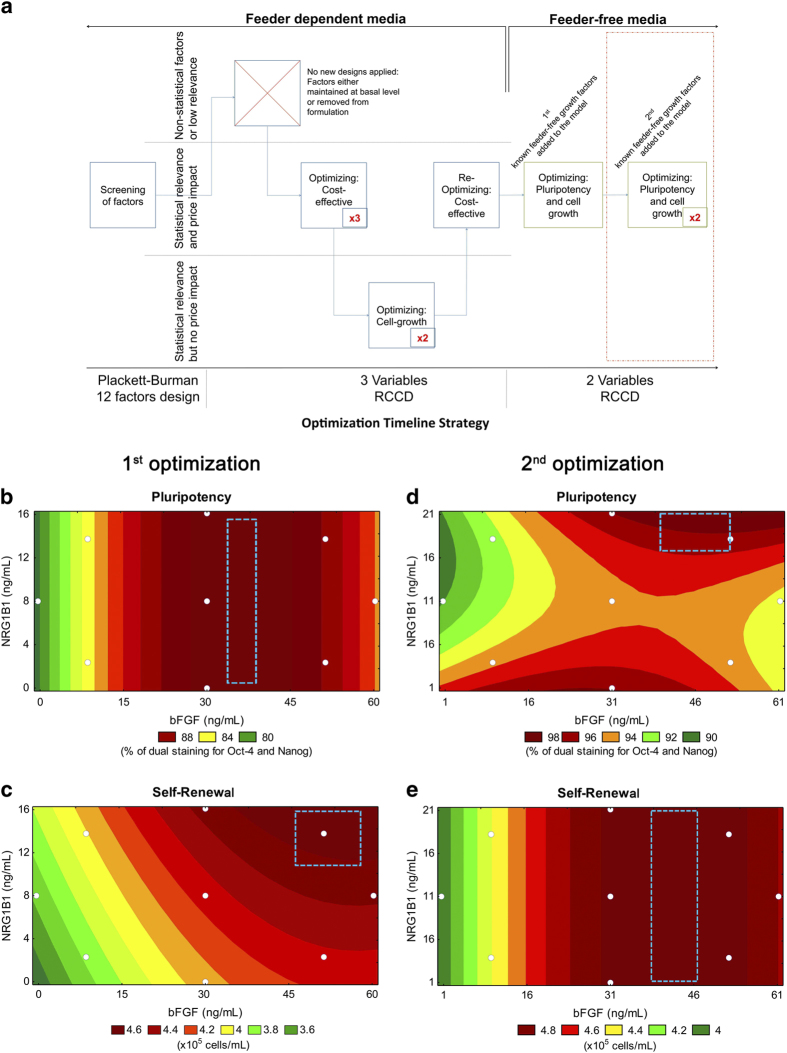
**A model for hPSC media optimization using design of experiments.** (**a**) Schematics of the rational used on the development of a completely recombinant, xeno- and feeder-free media. Each box represents one independent design, varying from 2 to 12 different factors, each designed was repeated one to three times depending on the readout of the model (number inside the box). The area dashed in red represents the part of media optimization that is reported in this manuscript. The first optimization from this work was performed using 0 – 60 ng of bFGF/mL and 0 – 16 ng of NRG1β1/mL. The second optimization was performed using 1 – 61 ng of bFGF/mL and 1 – 21 ng of NRG1β1/mL. Mathematical models were used to analyze the results from either the dual staining of OCT4 and NANOG (**b**, **d**) or for final cell concentration (**c**, **e**) White dots in the graphics represent the actual conditions performed. Colors ranging from dark red to dark green represent the highest to the lowest value for pluripotency and self-renewal. The dashed area in each model represents the optimum range predicted by the model to maximize the readout.

**Figure 2:**
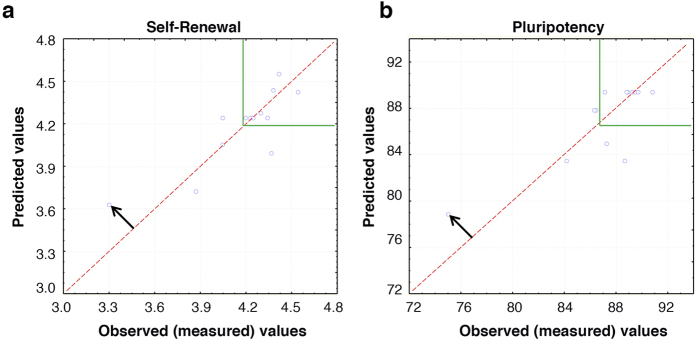
**Model predictability**. For each experimental condition, the observed (measured) value (blue dot) and the value predicted by the mathematical model for (**a**) self-renewal and (**b**) pluripotency, was shown. The less the distance between blue dot and the line of perfect predictability (red dashed line) is, the more the reliability of the model for that region is. The black arrows highlight the low predictability of the condition with lowest performance, in both cases, the experimental condition 5 (Table 1). However, in our region of interest (inside solid green lines), the distance between the line of perfect predictability and the blue dots inside that region is small in most of the cases. This confirms that the model can predict, in the range analyzed, the conditions with outstanding performance.

A 2-variable rotatable central composite design (2RCCD) was used to generate nine conditions (Table 1) allowing us to test bFGF from 0 to 60 ng/mL and NRG1β1 from 0 to 16 ng/mL. Each of the nine conditions was prepared in xeno-free basal medium that was previously optimized by our group (Supplementary Table 1) by several steps using different DOEs techniques^16^ (Fig. 1a). Efficacy was determined by measuring self-renewal (final cell concentration achieved) and pluripotency (dual positive staining for OCT4 and NANOG) of human embryonic stem cells (H9) using unbiased flow cytometry and automated cell counter. Although there are several ways to measure pluripotency, we choose these parameters because they are easy quantifiable read-outs. Further confirmation of pluripotency using other methods was tested on our final formulation (see below). The linear, quadratic and synergetic effects of each factor were generated (Table 2) and statistically relevant parameters that characterize self-renewal and pluripotency were used to make response surfaces (Fig. 1b,c). The pluripotency surface predicted that the optimum condition of bFGF was 35–45 ng/mL but found no effect based on the concentration of NRG1β1 (Fig. 1b). The self-renewal surface predicted the optimum conditions were 50 ng/mL bFGF and 16 ng/mL NRG1β1 (Fig. 1c). However, a better readout could be expected by extrapolating the up range of NRG1β1 value. Both surfaces fit the data reasonably well (R^2^ = 0.70). The fit between the observed effects and the model were weakest in regions of low pluripotency and self-renewal, which are regions of less interest (Fig. 2).

One potential shortcoming of this model is that bias might be introduced by the conditions entirely lacking in either bFGF or NRG1β1. To address this, a second set of experiments was performed where the minimum value tested for each factor was 1 ng/mL. In addition, the maximum value for NRG1β1 was increased from 16 ng/mL to 21 ng/mL based on extrapolations from the previous model. Again, a 2RCCD design (Table 1) was used to determine the test conditions and two readouts – pluripotency and self-renewal – were used to generate a second model (Table 2). In this model, maximal pluripotency was observed near 51 ng/mL of bFGF and 21 ng/mL of NRG1β1 (Fig. 1d). Maximal self-renewal was observed at 45 ng/mL of bFGF regardless of the concentration of NRG1β1 (Fig. 1e). Although self-renewal showed a poor correlation (R^2^ = 0.51), pluripotency was a better fit (R^2^ = 0.72). Taken together, both models support the choice of 51ng/mL of bFGF and 21 ng/mL of NRG1β1 for the formulation of an optimized media for self-renewal and pluripotency, which we refer to as iDEAL (Supplementary Table 2).

### iDEAL improves hPSC culture maintenance

**Figure 3:**
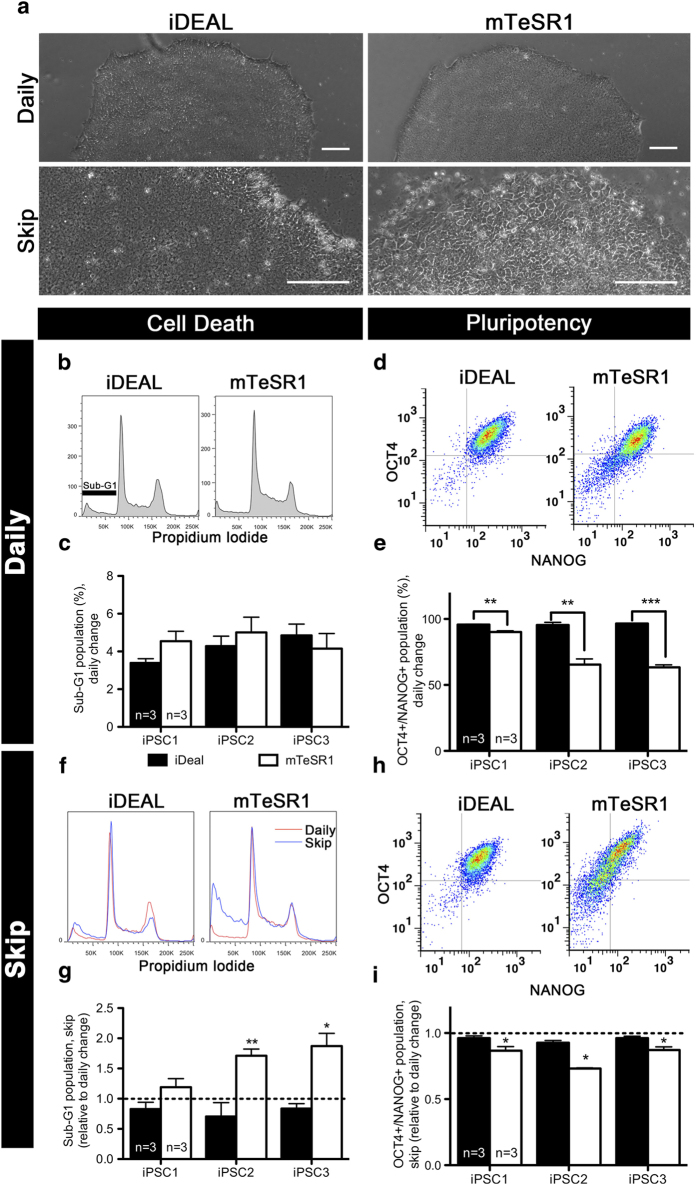
**The optimized iDEAL medium increases pluripotency of hPSC.** (**a**) Representative images of iPSCs passage 20 derived in iDEAL or mTeSR1 when the media were changed daily (top), or skipped on day 5 and 6 (bottom). Scale bar, 200 μm. (**b**) Representative cell cycle profiles of iPSC assessed by flow cytometry at day 7 after passaging when media were changed daily. (**c**) Sub-G1 population (%) at day 7 after passaging when media were changed daily. (**d**) Representative expression of pluripotent markers OCT4 and NANOG assessed by flow cytometry at day 7 after passaging when media were changed daily. (**e**) iPSC population (%) with OCT4 and NANOG expression at day 7 after passaging when media were changed daily. (**f**) Representative cell cycle profiles of iPSC at day 7 after passaging when media were skipped on day 5 and 6. (**g**) Change in sub-G1 population at day 7 after passaging when media were skipped on day 5 and 6. For each media type, sub-G1 iPSC population (%) in skipped condition was normalized to that in daily condition. (**h**) Representative expression of OCT4 and NANOG at day 7 after passaging when media were skipped on day 5 and 6. (**i**) Change in OCT4+and NANOG+population at day 7 after passaging when media change was skipped on day 5 and 6. For each media type, iPSC population (%) with OCT4 and NANOG expression in skipped condition was normalized to that in daily condition. Black bars represent iPSC derived in iDEAL, White bars represent iPSC derived in mTeSR1. For (**c,e,g,i**), data were shown as mean ± s.e.m for each of 3 iPSC lines (one clone each). n (technical replicates)=3. *P < 0.05, **P < 0.01, ***P < 0.001, two-sided unpaired Student’s *t* test.

To determine whether iDEAL improves the maintenance of pluripotency in hPSCs, we generated 3 lines of induced pluripotent stem cells (iPSC1, iPSC2 and iPSC3) from isolated female human fibroblasts using the methods described elsewhere^17^. After manual isolation from mouse embryonic fibroblasts (MEFs), iPSCs were cultivated without feeder cells using iDEAL or mTeSR1 as culture medium and Matrigel as extracellular matrix. Number of iPSC clones and technical replicates for each experiment were summarized in Supplementary Table 3. Similar to mTeSR1, all iPSCs derived in iDEAL expressed pluripotent markers, were able to differentiate into 3 germ layers *in vitro* and *in vivo* (teratomas), and maintained a normal karyotype (Supplementary Fig. 1). Late passage karyotyping analyses were also performed in different cell lines, as shown in our published patent^16^. After 7 days in culture with media changed on a daily basis, iPSCs (passage 20) derived in iDEAL or mTeSR1 are morphologically identical (Fig. 3a, top row) and have a similar percentage of cells undergoing apoptosis (sub-G1 phase population) as assessed by propidium iodide staining followed by flow cytometry^18^,^19^ (Fig. 3b,c). Regardless of the slightly intrinsic variations of different G1, S and G2/M populations between lines (Supplementary Fig. 2), it is undeniable that iDEAL maintained pluripotency better than mTeSR1, as indicated by a significantly (P = 0.0035 for iPSC1, P = 0.0033 for iPSC2 and P < 0.0001 for iPSC3, two-sided unpaired Student’s t test) higher percentage of double OCT4- and NANOG-positive cells (Fig. 3d,e). It was notable that even when the media was changed daily, approximately 60–70% of the cells remained pluripotent when cultured in mTeSR1, a finding we attribute to the result of unbiased passaging where differentiated cells are not removed prior to passaging (see Methods). In contrast, iDEAL cultures did not accumulate these differentiated cells.

The use of mTeSR1 for culturing iPSCs is both expensive and labor intensive as the cells need to be fed everyday due to their high metabolic activities^6^. To compare the two, we introduced a stressful condition by not changing the media on day 5 and 6 after passaging and then determined pluripotency and cell death on day 7. Whereas iPSC colonies in iDEAL appeared to be morphologically normal (Fig. 3a, bottom left), iPSCs in mTeSR1 showed morphological alterations at the edge of colony (Fig. 3a, bottom right). iPSCs in iDEAL had few sub-G1 cells (Fig. 3f, left and g, black bar) and maintained expression of both OCT4 and NANOG (Fig. 3h, left and i, black bar). In contrast, cells cultured in mTeSR1 had a significantly larger population of sub-G1 cells (Fig. 3f, right and 3G, white bar, *P* = 0.0046 for iPSC2 and *P* = 0.0118 for iPSC3, two-sided unpaired Student’s *t* test) and decreased expression of both OCT4 and NANOG (Fig. 3h, right and i white bar, *P* = 0.0146 for iPSC1, *P* = 0.0155 for iPSC2 and *P* = 0.0268 for iPSC3, two-sided unpaired Student’s *t* test). These results suggest that iDEAL maintains iPSC pluripotency and minimized cell death more efficiently than mTeSR1.

### iDEAL facilitates single hPSC passaging

**Figure 4:**
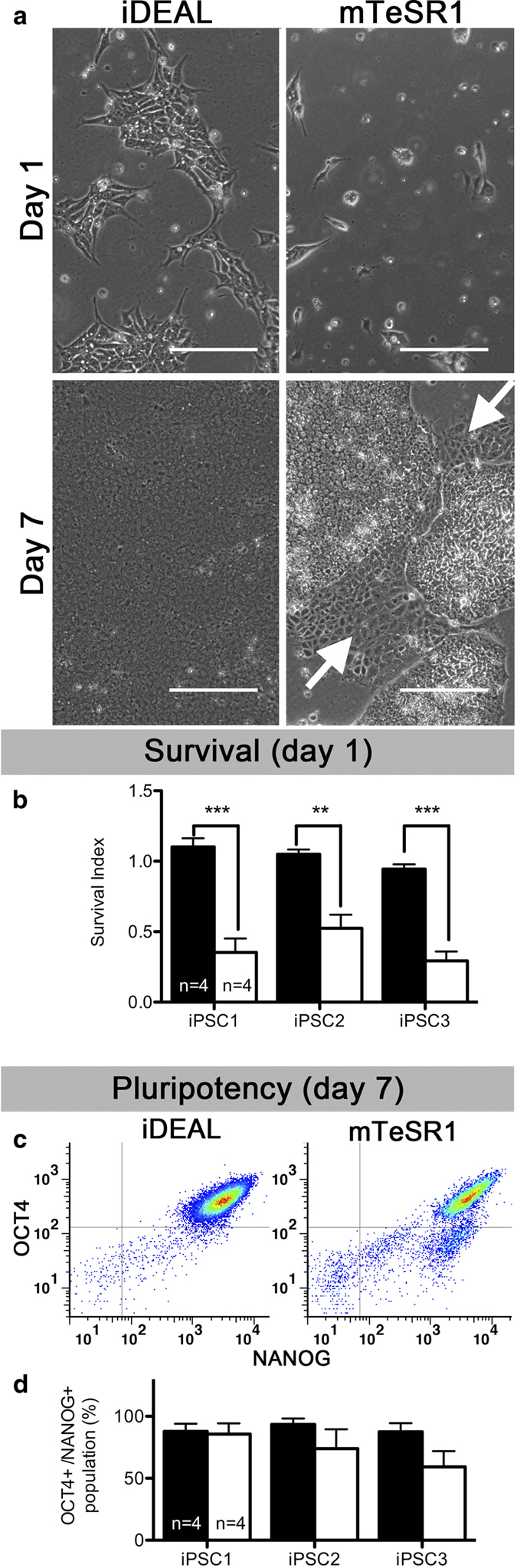
**The optimized iDEAL medium improves single-cell survival of hPSC.** (**a**) Representative images of iPSCs passage 20 plated as single cells in iDEAL or mTeSR1 at day 1 and at day 7. Arrows point to differentiated cells. Scale bar, 200 μm. (**b**) Survival index of iPSCs at day 1 after plating calculated by dividing number of live cells at day 1 by number of cells plated on day 0. (**c**) Representative expression of pluripotent markers OCT4 and NANOG assessed by flow cytometry at day 7 after plating as single cells in each medium. (**d**) iPSC population (%) with OCT4 and NANOG expression at day 7 after plating as single cells. Black bars represent iPSC derived in iDEAL, White bars represent iPSC derived in mTeSR1. Data were shown as mean ± s.e.m for each of 3 iPSC lines (one clone each). n (technical replicates)=4. **P < 0.01, ***P < 0.001, two-sided unpaired Student’s *t* test.

hPSC are typically passaged as small clumps and not as single cells in order to keep them viable and undifferentiated^20^,^21^. This practice though makes hPSC maintenance much more tedious and time consuming as compared to the maintenance of other cell types such as fibroblasts and neural progenitor cells where complete dissociation during passaging is feasible. We determined whether iPSCs derived in iDEAL could survive single-cell plating while maintaining pluripotency. When the same number of iPSCs dissociated into single cells was plated in either iDEAL or mTeSR1, with the addition of 10 μM Rock inhibitor (Supplementary Fig. 3a), the number of live cells in iDEAL 1 day later were significantly (P = 0.0007 for iPSC1, P = 0.002 for iPSC2 and P = 0.0001 for iPSC3, two-sided unpaired Student’s t test) higher than in mTeSR1 (Fig. 4a, top row, and b). Moreover, since dissociation could induce the differentiation^22^, we also measured the pluripotency of surviving cells. Although differentiated cells were clearly observed in mTeSR1 culture (Fig. 4a, bottom right) and not in iDEAL culture (Fig. 4a, bottom left) 7 days after plating, the percentage cells expressing both NANOG and OCT4 as assessed by flow cytometry was not significantly lower in mTeSR1 culture (Fig. 4c,d). iDEAL-derived iPSCs that were passaged as single cells for 5 passages remained highly positive for both NANOG and OCT4 (Supplementary Fig. 3b) and were capable of forming EBs (Supplementary Fig. 3c), suggesting that pluripotency of iDEAL-derived iPSCs were maintained after several single-cell passaging. These results indicate that iDEAL would be a better choice for iPSC single-cell passaging, as it is simpler and faster than traditional clump passaging.

### Effect of iDEAL on X chromosome inactivation (XCI) status of iPSCs

**Figure 5:**
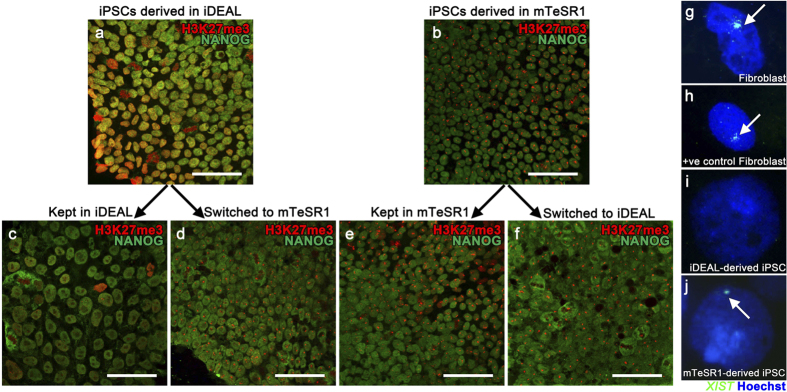
**H3K27me3 mark in iPSCs is influenced by culture media.** Immunostaining profile of H3K27me3 (puncta) and NANOG and *XIST* expression on iPSCs derived in different media. Cells derived in iDEAL medium (**a**, **c**, **d**) and in mTeSR1 medium (**b**, **e**, **f**). Immunostaining was performed at the establishment of the line (A, B), passage 4, and after long-term maintenance (**c**, **e**), passage 10, with media swap from passages 7 to 10 (**d**, **f**). Scale bar, 100 μm. *XIST* RNA FISH was performed in fibroblast before reprogramming (**g**), positive control fibroblast (**h**), iPSC derived in iDEAL (**i**) and iPSC derived in mTeSR1 (**j**).

The mechanism of reactivation of X chromosomes during reprogramming is not fully understood^23^, limiting the potential of iPSCs for X-linked diseases^24^. It has been shown recently that the addition of recombinant leukemia inhibitory factor (LIF) to the hPSC culture can reactivate inactive X chromosome observed in early passage^25^, suggesting that media formulations can influence the status of X chromosome. In order to test the hypothesis that iDEAL could influence the X-inactivation status during the derivation of iPSCs, we measured the epigenetic marker trimethylated histone 3 lysine 27 (H3K27me3) in iPSCs derived in mTeSR1 or iDEAL. H3K27me3 marks silenced DNA including the inactive X chromosome in interphase nuclei^26^,^27^. This mark has been widely used to discriminate the status of X chromosomes in human pluripotent stem cells^17^,^28^,^29^. Half of the iPSC clones derived in iDEAL did not generated H3K27me3 staining foci in early passages (Fig. 5a). However, all iPSC clones derived in mTeSR1 had a very clear positive staining for H3K27me3 (Fig. 5b). Similar results were found for cells after >10 passages thereby ruling out the possibility that iDEAL simply resulted in a delayed transitory state (Fig. 5c). The absence of H3K27me3 marks in late passage of iPSCs could be caused by erosion of XCI^24^ or by the loss of those marks even though the X chromosome remains inactive^30^. To test for these possibilities, we performed a media swap experiment and found that the H3K27me3 mark could be detected after changing from iDEAL to mTeSR1 (Fig. 5d). This suggests that the absence of H3K27me3 was not due to either erosion of XCI or the loss of XCI marks in inactive X chromosome. Moreover, these studies allowed us to determine if iDEAL was able to reset this epigenetic marker. Similar to iPSCs derived and maintained in mTeSR1 (Fig. 5e), we found that iPSC clones initially cultured in mTeSR1 retained H3K27me3 foci when transferred to iDEAL (Fig. 5f). This suggests that rather than controlling the presence of H3K27me3, iDEAL maintained the absence status of this marker once it was set upon reprogramming. We validated these observations by performing fluorescent *in situ* hybridization (FISH) using the XIST RNA expression, another marker for inactive X chromosomes^31^. Whereas XIST RNA was clearly expressed in fibroblasts before reprogramming (Fig. 5g), positive control (Fig. 5h) and fibroblast-derived iPSCs in mTeSR1 (Fig. 5j), it was not detected in iPSCs derived in iDEAL (Fig. 5i). This result suggests that the medium formulation can affect the X activation status. It is important to note that 3 out of 6 clones from one iPSC line derived in iDEAL showed no H3K27me3 mark for entire population, and one clone showed mixed population for this marker. Nonetheless, our findings, consistent with those of others, demonstrate the importance of culture medium on epigenetic markers involving X inactivation in female iPSCs.

## Discussion

The striking advantage of DOE over other empirical methods in the optimization of multiple parameters is that the most optimal condition could be mathematically and statistically determined when sufficient conditions are tested. We introduced this strategy to pluripotent stem cell research and showed its practical application by using hPSC culture media as an example. We developed the final media for hPSC, iDEAL in feeder-free Matrigel based system, by optimizing the final concentration of 2 empirically established factors; bFGF and NRG1β1. While bFGF is long known as major factor keeping pluripotent stem cells in undifferentiated state^32^,^33^, NRG1β1 has been proved to be another important player for less than 10 years^15^. For maintaining hPSC in feeder-free system, many groups have claimed best concentration of bFGF at 100 ng/mL^34^,^35^ for over decades and of NRG1β1 at 10 ng/mL^15^. Regular methods to define best concentrations in hPSC field usually starts with random dictates of the concentration developed around the previously known values, followed by the test of each concentration^34^,^36^. The one with the best readouts, e.g. lowest apoptotic and highest proliferation rate, was selected and claimed as the most optimal concentration that should be used to maintain hPSC culture. This empirical method has two major drawbacks. First, the true ‘best concentration’ could possibly fall in the untested interval between the ones chosen by experimenters. Second, since those studies optimized the concentration of one factor at a time, the interaction between these factors that could affect the readouts is neglected. This is the case for the pluripotency E8 media, while some of their discovers are extremely relevant as the toxic influence of the β-mercaptoethanol (BME) in albumin-free media^37^ as our group also demonstrated few years ago in a dose-dependent manner^16^, other hypothesis for optimizing the media are simply removing few components from mTeSR1 media instead of understanding their real contribution. For instance, we have identified that the optimum supplementation of non-essential amino acids (NEAA), glutamine and citric acid on a DMEM/F12 based medium could increase cell proliferation by 20-30%^16^. In contrast to mTeSR1 or iDEAL, the E8 media do not use NEAA supplementation as^38^,^39^, it was just removed without proper measurement of its effect the overall formulation. The same is also valid for other factors, such as serum derived albumin. While the concerns of inter-batch variability from serum-derived components is undeniable, options of recombinant proteins with insignificant batch-to-batch variation are already available and therefore could be used as option to improve the media in a cost-effective fashion, as it was done in iDEAL pre-development^16^. The pluripotency and number of cells from each condition tested were used to mathematically generate the model and find the most optimal concentration of these factors. For NRG1β1, the first optimization suggested that 12 ng/mL or higher should be used to keep both pluripotency and self-renewal at maximal performance. In the second optimization conducted to validate that the absence of these factors did not bias the model, any concentration resulted in similar percentage of self-renewal. However, this is not the case for pluripotency where only high concentration must be used to maintain high percentage of pluripotency. Hence, to satisfy both pluripotency and self-renewal at highest efficiency, only high range of NRG1β1 must be used in the formulation. Our results indicate that iDEAL could contribute to many aspects of stem cell research.

As hPSC work routine requires daily feeding, the ideal concept of weekend-free hPSC culture has been developed for a while. In general, since the cell density on the day after passaging (day 1) is low and thus the metabolic activity is not as high, the feeding could be omitted on the day after passaging. By adding extra media to the culture on the day passaging is done (day 0) as commercially suggested, it is expectedly feasible to let the cells stay in the same environment for another day (continuously omitted on day 1 and day 2). In other words, weekend-free hPSC culture is possible only if the passaging is done on Friday. In our study, to more extreme extent, we skipped the media change on day 5 and 6 after passaging when the cells are almost confluent and iDEAL still maintained the level of cell death and pluripotency as if the cells were fed daily. We speculate that the difference in apoptosis between the two systems is likely due to the fact that that empirically formulated medium (mTeSR1) was not truly flexible to accommodate stressful conditions, limiting the range of tolerance for hPSC in this system. On the other hand, systematically optimized medium (iDEAL) can better buffer the cellular stress, presumably by inducing intrinsic epigenetic modifications in a subset of genes/pathways responsible for this flexibility. Thus, we prove that iDEAL supports the weekend-free culture regardless of the day the passaging performed, allowing more flexible work routine.

While small amount of spontaneous differentiation in hPSC culture is expected and signifies that these cells are capable of differentiating into other cell types, excessive amount could be a problem as it could interfere with culture maintenance as well as an intended differentiation. Several published protocols recommend removing differentiated cells from hPSC culture before passaging^21^,^39^,^40^,^41^,^42^,^43^,^44^. Such time- and labor-consuming step is not required when iPSCs are in iDEAL system; most likely this could be related to the fine optimization of growth factors known to interfere in pluripotency such as FGF-2 and NRG1β1, the last one absent in mediums like mTeSR1 or E8. In our study, iPSC culture in mTeSR1 accumulated differentiated cells over passages as shown by lower percentage of pluripotency compared to culture in iDEAL at the same passage number. One might argue that difference between cell lines themselves could presumably contribute to the level of pluripotency as well as subG1 population^45^. While this seems to be the case for mTeSR1 culture as the variation was clearly observed between iPSC lines, the discrepancy was greatly reduced when cells from the same subjects were in iDEAL culture condition, implying that iPSCs in iDEAL are in more naïve state, the ideal scenario one would like to have in hPSC culture.

Single-cell plating of hPSC has been pursuing in the field but never been efficient either because of low survival of hPSC after complete dissociation^21^ or the requirement of several extra factors added to culture medium^46^. However, our optimized medium supplemented with ROCK inhibitor only on day 0 overcomes these 2 obstacles. In a pilot single-cell plating experiment, we did not add ROCK inhibitor to the culture when plating single iPSCs. Unlike iDEAL-derived iPSCs, mTeSR1-derived iPSCs plated without ROCK inhibitor had a very low percentage of survival after single-cell plating and took unusually long time to recover, making the comparison between two media non reliable. To have sufficient mTeSR1-derived iPSCs for the analysis and allow for comparison with iDEAL-derived iPSCs in timely fashion, we added ROCK inhibitor to both medium systems when we plated them (day 0 only). Although we have not looked further than survival and pluripotent marker expression of plated iPSCs after complete dissociation, the readouts are sufficient enough to show the vast difference between the cells cultured in each media. The survival of hPSC in iDEAL is doubly improved from that in mTeSR1 while maintaining the pluripotency at the same level. This complete dissociation method combined with iDEAL could be adapted as routine hPSC passaging, hugely decreasing time spent carefully breaking down hPSC colonies and facilitating scalable and controllable culture for industrial and clinical uses as the passaging protocol becomes as simple as the one for routine fibroblast passaging. Moreover, this system could also potentially facilitate particular technique such as plasmid transfection in hPSC via electroporation. The more the single iPSCs survive, the greater the transfection efficiency is obtained. Further analyses are needed to verify that quality of hPSCs passaged as single cells in iDEAL is similar to that of hPSCs passaged as clumps.

The inconsistency of XCI status in hiPSCs has been reported^47^. While most groups generated hiPSCs retaining one inactive X chromosome^48^,^49^,^50^,^51^, a few showed that inactive X chromosome in somatic cells is reactivated upon reprogramming^17^,^52^. Although it is unclear how reactivation of X chromosome upon reprogramming could be manipulated in the culture, several studies found that the derivation and culture conditions, such as oxygen levels^53^ and persistency of pluripotent factor expression^54^, could influence the status of the X chromosome. Medium composition has been shown to reactivate inactive X chromosome in iPSC at a few passages after reprogramming^25^. Using iDEAL, we were also able to derive iPSCs with both active X chromosomes with at least 50% efficiency although the results were based on only the absence of H3K27me3 and of XIST RNA expression. When studying expression of X-linked genes and XCI status upon differentiation, iDEAL could be an attractive tool to reactivate X chromosome upon reprogramming.

## Conclusion

We have developed a systematically optimized hPSC media, iDEAL. By using DOE, we are able to manipulate and discover the most effective combination of bFGF and NRG1β1 that robustly improves culture condition for hPSC. DOE could also be further applied to improve numerous non-fully defined or non-optimal conditions in hPSC field, such as differentiation protocols to obtain specific cell types that have low differentiation efficiency or reprogramming conditions. The whole optimization of iDEAL formulation has several advantages over empirically-formulated media. By maintaining a higher percentage of pluripotent cells, the use of iDEAL could decrease the vast amount of time spent removing unwanted differentiated cells typically present in iPSC cultures. The use of iDEAL also results in cultures with increased viability, even if the media is not changed for two days. Moreover, iDEAL improves the survival of iPSCs when plated as single cells, which could be very useful for routine passaging and several assays including transfection and drug screening, such as for genome editing. Finally, our data strongly suggest that hPSC media can influence epigenetic markers and X inactivation. Further improvements on our formulation will maximize the use of iDEAL in assays requiring consistency of X-chromosome inactivation status. More broadly, these findings show that a mathematical modeling approach can be used to solve sophisticated biological problems by systematic optimization of numerous different factors. Our data strongly support the need of systematical optimization of hPSC culture conditions and similar strategy can be used for hPSC differentiation protocols.

## Materials and Methods

### Mathematical Modeling and Statistical Analysis

The methodology for the medium optimization using H9 cells consisted of a 2-variable rotatable central composite design (2RCCD). Mainly, two readouts were used in order to evaluate self-renewal and pluripotency of H9 cells. The first one, self-renewal, was obtained through the automated counting of the cell concentration after at least 6 days of culture. Pluripotency was defined as the percentage of the population double-stained for NANOG and OCT4 at the end of the culture. The software Statistica 7.0 (StatSoft, USA) was used in order to generate the matrixes which contained the specification of each medium condition formulation, accordingly to the variation created for every supplement for the concentration range set. In brief words, the software calculates the effects of each supplement for every readout, self-renewal or pluripotency, by utilizing the least square method. The error of each sample is estimated from the error obtained by the genuine replicates performed on the central points of the matrix, condition number 9, done at least in 4 independent experiments. Afterwards, the software is able to generate the relevance of each factor by comparing the ratio of the calculated effects and its errors with the bilateral Student’s t-distribution with the proper degree of freedom. Then, the p-value is obtained for each parameter of the model. A conservative approach was adopted and all parameters with a p-value < 0.1 were considered statistical relevant for the mathematical model. Note that a probability normal plot was performed and samples followed a linear straight line, therefore, suggesting a normal distribution. No “S” shapes, characteristics from bimodal distributions, and also, no breaks in any part of the range analyzed, that could suggest other abnormalities, were identified.

### Human embryonic stem cell culture

H9 cells were obtained from Human Stem Cell Core Facility, UCSD, and tested negative for mycoplasma contamination by PCR. They were cultured in feeder-free condition using Matrigel (~100 μg/mL, BD Biosciences) as basement membrane matrix, fed with mTeSR1 (Stemcell Technologies) or different formulations of medium on daily basis and passaged enzymatically weekly.

### Reprogramming and iPSC maintenance

The study protocols were approved by IRB/ESCRO committee review (IRB#100574ZF). Female human primary fibroblasts were obtained with informed consent from subjects and tested negative for mycoplasma contamination by PCR. The fibroblasts were reprogrammed and characterized using the method previously described elsewhere^17^. Upon manual isolation from MEFs, iPSCs were grown on Matrigel-coated dish in either iDEAL (see Supplementary Table 2 for complete composition) or mTeSR1 (Stemcell Technologies). For media change and single-cell plating experiments, one clone for each line (3 lines in total) was used. For X chromosome inactivation experiments, six clones of one line were used. Summary of clones and technical replicates for each experiment could be found in Supplementary Table 3. Medium was changed daily. iPSCs were passaged semi-enzymatically on weekly basis. Briefly, cells were incubated in calcium- and magnesium-free PBS for 5 min and lifted using cell scraper. After partial dissociation by up-and-down pipetting, cells were plated onto new Matrigel-coated plate. Note that no removal of differentiated cells was done prior to the passaging.

### Embryoid body (EB) formation and RNA extraction

70-90% confluent iPSCs (day 6-7 after passaging) were fed with N2 medium (1X N2 supplement (Life Technologies) in DMEM/F12 (Cellgro)) for 2 days. On the next day, iPSCs were treated with calcium- and magnesium-free PBS for 5 min and lifted using cell scraper. Next, iPSCs were transferred to one well in 6-well plate in N2 medium supplemented with 1% fetal bovine serum (Gemini Bio-Products), and cultured on shaker at 37 °C. Medium was replaced every 3 days. After 20 days on the shaker, RNA extraction from EB was performed using Trizol® (Life Technologies) according to manufacturer protocol.

### Teratoma formation assay

iPSC colonies from 2 confluent 100 mm dishes were harvested after treatment with StemPro® Accutase® (Life Techonologies), pelleted, and suspended in 300 μL Matrigel. The cells were injected subcutaneously into nude mice; 6 to 8 weeks after injection, teratomas were dissected, fixed overnight in 10% buffered formalin phosphate, and embedded in paraffin. The sections were stained with hematoxylin and eosin for further analysis. The protocols were approved by the University of California San Diego Institutional Animal Care and Use Committee.

### Media change experiments

iPSC derived in either iDEAL or mTeSR1 were passaged semi-enzymatically as described above and seeded onto Matrigel-coated 6-well plate at density of 1 × 10^5^ cells/well . Media change was done on a daily basis from day 1 to 6 for ‘Daily’ condition. For ‘Skip’ condition, feeding was done on a daily basis from day 1 to 4 and omitted on day 5 and 6. On day 7, iPSCs were completely dissociated using Stempro® Accutase® (Life technologies) and collected; one half was used for cell death analysis and the other for pluripotency analysis.

### Cell death analysis

iPSCs collected at day 7 were fixed using cold absolute ethanol overnight at 4 °C. After 2 washes with PBS, cells were incubated in staining solution (20 μg/mL of propidium iodide and 200 μg/mL of RNase in PBS) for 30 min at room temperature. Flow cytometry was done on a FACSCanto™ II (BD Biosciences) and analyzed using Flowjo (Tree Star, Inc). Sub-G1 population was used to calculate the following parameters; (1) percentage of cell death in ‘Daily’ condition, (2) increase in cell death in ‘Skip’ condition over corresponding ‘Daily’ condition.

### Pluripotency analysis

iPSCs collected were fixed with 4% paraformaldehyde in PBS for 15 min and washed with PBS for 3 times, 5 min each. Cells were then permeabilized and blocked with 0.1% triton X and 2.5% bovine serum albumin for 30 min and incubated with primary antibodies (mouse anti-OCT4 from Santa Cruz (cat#SC-5279), 1:250; rabbit anti-NANOG from GeneTex (cat#GTX100863), 1:400) for 30 min at 4 °C. After washed with PBS for 3 times, cells were incubated with secondary antibodies (Alexa Fluor 488 donkey anti-mouse IgG (cat#A21202), 1:500; Alexa Fluor 555 donkey anti-rabbit IgG (cat#A31572), 1:500, from Life Technologies) for 30 min at 4 °C. After washed, flow cytometry was done on LSRFortessa (BD Biosciences) and analyzed using FACSDIVA (BD Biosciences). Every sample was used for the analysis. For media change experiment, population with dual staining (both OCT4 and NANOG positive) was used to calculate the following parameters; (1) percentage of pluripotent cells in ‘Daily’ condition, (2) reduction in pluripotent cells in ‘Skip’ condition over corresponding ‘Daily’ condition. For single-cell plating experiment, population with dual staining (OCT4 and NANOG) was used to calculate the percentage of cells remaining pluripotent at day 7 after plated as single cells.

### Single-cell plating

iPSCs at 80% confluency were completely dissociated after incubation with Stempro® Accutase® (Life technologies) for 5 min. Cells were passed through 40 μm cell strainer to ensure the singularity (Supplementary Fig. 2), counted using Trypan Blue exclusion assay and then seeded onto Matrigel-coated 6-well plate at density of 2 × 10^5^ cells/well (day 0). ROCK inhibitor (10 μM, R&D system) was added with the media only on day 0 to improve the survival^20^. Media were then changed daily. Cells were collected at day 1 for survival analysis and at day 7 for pluripotency analysis.

### Survival analysis

At 24 hours after single-cell plating, cells were collected, completely dissociated using Stempro® Accutase® (Life technologies) and counted using Trypan Blue exclusion assay. Survival index was determined by dividing number of live cells collected at day 1 by number of cells seeded on day 0 (2 × 10^5^ cells).

### Statistical analysis for pluripotency, cell death and survival

Mean ± s.e.m. for each parameters mentioned above were obtained from 3 and 4 independent technical replicates of one clone for each of 3 iPSC lines for media change and single-cell plating experiments, respectively. There were no adjustments for multiple comparisons. To statistically compare the means of two unmatched groups where normal distribution and similar variance between groups was statistically confirmed, two-sided unpaired Student’s t-test was used and significance was defined as P < 0.05.

### Immunofluorescence staining

iPSCs were fixed with 4% paraformaldehyde in PBS for 15 min and washed with PBS for 3 times, 5 min each. Cells were then permeabilized and blocked with 0.1% triton X and 2.5% bovine serum albumin in PBS for 1 hour and incubated in primary antibodies overnight at 4 °C (mouse anti-OCT4 from Santa Cruz (cat#SC-5279), 1:250; rabbit anti-LIN28 from Abcam (cat#AB46020), 1:500; mouse anti-SSEA4 from Abcam (cat#MC813), 1:200; rabbit anti-NANOG from GeneTex (cat#GTX100863), 1:300; goat anti-NANOG from R&D (cat#AF1997), 1:250; rabbit anti-H3K27me3 from Millipore (cat#07-449), 1:500); mouse anti-Tra-1-60 from Abcam (cat#AB16288), 1:500; and anti-Tra-1-81 Alexa647 from BD Biosciences (cat#BDB560124), 1:10). After washed with PBS for 3 times, 5 min each, cells were incubated in secondary antibodies if necessary (Alexa Fluor 488 donkey anti-mouse IgG (cat#A21202), 1:500; Alexa Fluor 555 donkey anti-rabbit IgG (cat#A31572), 1:500; Alexa Fluor 488 donkey anti-goat IgG (cat#A11055), 1:500, from Life Technologies) for 1 hour at room temperature and nuclei were stained using DAPI (1:5000).

### X inactivation (media swap) experiments

iPSCs derived in either iDEAL or mTeSR1 were continuously cultured in its original medium until passage 6. At passage 7, iPSCs derived in iDEAL were split into two culture conditions; one was still fed with iDEAL and the other was fed with mTeSR1 until passage 10. The same strategy was applied to iPSCs derived in mTeSR1. Immunofluorescence staining against H3K27me3 (described above) was performed in iPSCs at passage 4 and 10 of all conditions. Images were obtained using an Olympus Fluoview FV1000 confocal microscope.

### Fluorescence *in situ* hybridization (FISH)

iPSCs were grown in 25cm^2^ tissue culture flask until 60-70% confluency. FISH for XIST were performed by Molecular Diagnostic Services (San Diego, CA)^39^.

## Author Contributions

P.M. designed the experiments, optimized and validated the medium formulation in H9 cells, performed X inactivation experiments, and wrote the manuscript. T.C. derived and characterized iPSC lines, performed media change, single-cell plating and X inactivation experiments and wrote the manuscript. A.R.M provided financial and environmental support, designed the experiments and edited the manuscript.

## Additional Information

**How to cite this article**: Marinho, P. A. *et al*. Systematic optimization of human pluripotent stem cells media using Design of Experiments. *Sci. Rep.* 5, 09834; doi: 10.1038/srep09834 (2015).

## Supplementary Material

Supporting Information Supplementary Figures 1-3 and Supplementary Tables 1-3

## Figures and Tables

**Table 1:** Concentration of the factors in each experimental condition evaluated for the 2 independent 2RCCDs.

**Conditions**	**bFGF**	**NRG1β1**
1	−1	−1
2	−1	+1
3	+1	−1
4	+1	+1
5	−1.41	0
6	+1.41	0
7	0	−1.41
8	0	+1.41
9	0	0
Concentrations on 1^st^ model	μg/L	μg/L
+1.41	60	16
+1	51.2	13.6
0	30	8
−1	8.8	2.4
−1.41	0	0
Concentrations on 2^nd^ model	μg/L	μg/L
+1.41	61	21
+1	52.2	18.1
0	31	11
−1	9.8	3.9
−1.41	1	1

Nine conditions were created in a matrix according to 2RCCD. The un-coded values of each number in the matrix are showed below for every model and factor.

**Table 2:** Effects and statistical relevance of each mathematical parameter in the 2RCCDs.

**1**^**st**^ **2RCCD**						
**Self-renewal**	**Coefficient**	**Error**	**t (4)**	**p-value**	**Min (90%)**	**Max (90%)**
Mean	4.21	0.05	89.32	0.00	4.11	4.31
bFGF (Linear)	0.28	0.04	7.49	0.00	0.20	0.36
bFGF (Quadratic)	−0.10	0.04	−2.60	0.06	−0.19	−0.02
NRG1β1 (Linear)	0.14	0.04	3.64	0.02	0.06	0.22
NRG1β1 (Quadratic).	0.04	0.04	1.09	0.34	−0.04	0.13
bFGF x NRG1β1	−0.09	0.05	−1.80	0.15	−0.21	0.02
Pluripotency	Coefficient	Error	t (4)	p-value	Min (90%)	Max (90%)
Mean	89.18	0.61	145.94	0.00	87.88	90.48
bFGF (Linear)	2.16	0.48	4.48	0.01	1.13	3.19
bFGF (Quadratic)	−3.69	0.52	−7.12	0.00	-4.79	-2.59
NRG1β1 (Linear)	-0.52	0.48	-1.07	0.34	-1.55	0.51
NRG1β1 (Quadratic).	0.29	0.52	0.55	0.61	-0.82	1.39
bFGF x NRG1β1	1.08	0.68	1.57	0.19	-0.38	2.53
**2**^**nd**^ **2RCCD**						
Self-renewal	Coefficient	Error	t (4)	p-value	Min (90%)	Max (90%)
Mean	4.90	0.13	38.76	0.00	4.63	5.17
bFGF (Linear)	0.27	0.10	2.72	0.05	0.06	0.49
bFGF (Quadratic)	−0.27	0.11	−2.51	0.07	−0.50	−0.04
NRG1β1 (Linear)	0.14	0.10	1.38	0.24	−0.08	0.35
NRG1β1 (Quadratic).	−0.12	0.11	−1.09	0.34	−0.35	0.11
bFGF x NRG1β1	−0.10	0.14	−0.72	0.51	−0.40	0.20
Pluripotency	Coefficient	Error	t (4)	p-value	Min (90%)	Max (90%)
Mean	93.78	0.41	230.59	0.00	92.91	94.65
bFGF (Linear)	0.94	0.32	2.93	0.04	0.26	1.63
bFGF (Quadratic)	−1.23	0.34	−3.56	0.02	−1.96	−0.49
NRG1β1 (Linear)	−0.14	0.32	−0.44	0.68	−0.83	0.54
NRG1β1 (Quadratic).	1.10	0.34	3.18	0.03	0.36	1.83
bFGF x NRG1β1	1.08	0.45	2.36	0.08	0.11	2.04

The linear, quadratic and synergetic effects of each factor are represented on the coefficient column. The statistical relevance (t) and the p-value were also calculated and every parameter with a p-value < 0.1 was considered statistically relevant (highlighted in red). The columns “Min” and “Max” represent the margin values of each parameter with 90% confidence.
