# Supplementary material for: Systematic optimization of human pluripotent stem cells media using Design of Experiments
Source: Sci Rep. 2015 May 5;5:9834. doi: 10.1038/srep09834 (PMC4419516; doi:10.1038/srep09834)

Ecological impacts of large-scale disposal of mining waste in the deep sea

David J. Hughes, Tracy M. Shimmield, Kenneth D. Black & John A. Howe

Supplementary Tables and Figures

Supplementary Table S1

Location and depth data for the benthic stations sampled off Lihir (L1-L6) and Misima (M1-M6), with the number of replicate corer drops used for biological sampling at each station.

Station	Latitude	Longitude	Depth (m)	No. corer drops
L1	03^{0} 06.200’S	152^{0} 40.540’E	850	7
L2	03^{0} 07.000’S	152^{0} 46.370’E	1750	5
L3	03^{0} 06.640’S	152^{0} 49.790’E	2020	6
L4	03^{0} 08.260’S	152^{0} 30.880’E	800	5
L5	03^{0} 00.360’S	152^{0} 26.070’E	1715	3
L6	02^{0} 56.901’S	152^{0} 22.266’E	2020	4
M1	10^{0} 44.500’S	152^{0} 51.242’E	1380	3
M2	10^{0} 46.184’S	152^{0} 54.356’E	1461	3
M3	10^{0} 47.280’S	152^{0} 56.210’E	1467	6
M4	10^{0} 50.000’S	152^{0} 49.000’E	1793	3
M5	10^{0} 58.561’S	153^{0} 40.943’E	1704	3
M6	10^{0} 56.425’S	152^{0} 23.503’E	1250	3

Supplementary Table S2

Content of selected metals in sediments from stations east (L1-L3) and west (L4-L6) of Lihir. Data show solid-phase inventories (g m^{-2}) summed over the 0-14 cm depth range for individual cores.

Station	Depth (m)	Ca	As	Cu	Pb	Cd
L1	850	8675.7	32.8	15.8	6.5	0.0
L2	1750	3647.4	33.1	19.6	9.3	0.1
L3	2020	16283.9	10.2	13.3	3.8	0.1
L4	800	22887.3	1.5	8.7	0.6	0.0
L5	1715	17577.7	1.2	5.4	0.7	0.0
L6	2020	16768.7	0.8	6.0	0.7	0.0

Supplementary Table S3

Higher-taxon composition of metazoan meiofaunal communities at stations east (L1-L3) and west (L4-L6) of Lihir. Data show mean (± SD) percentage total individuals (> 250 µm) at each station, using corer drops as replicate samples.

Station	Depth (m)	Nematoda	Copepoda	Ostracoda	Others
L1	850	20.9 ± 22.9	70.3 ± 28.6	4.8 ± 12.6	4.0 ± 7.0
L2	1750	2.4 ± 3.9	97.6 ± 3.9	-	-
L3	2020	16.7 ± 40.8	83.3 ± 40.8	-	-
L4	800	51.8 ± 8.9	40.0 ± 6.5	7.2 ± 3.2	1.0 ± 1.6
L5	1715	41.1 ± 13.1	50.8 ± 6.5	5.4 ± 5.3	2.5 ± 2.3
L6	2020	40.2 ± 8.0	49.2 ± 8.0	7.8 ± 3.2	2.9 ± 3.5

Supplementary Table S4

Content of selected metals in sediments from stations around Misima. Data show solid-phase inventories (g m^{-2}) summed over the 0-10 cm depth range for individual cores.

Station	Depth (m)	Ca	As	Cu	Pb	Cd
M1	1380	3937.7	1.4	18.0	55.5	0.8
M2	1461	3826.7	1.2	16.1	54.7	0.6
M3	1467	4262.4	1.1	16.7	60.0	0.5
M4	1793	14309.0	0.4	4.0	9.3	0.1
M5	1704	22189.1	0.2	1.7	1.8	0.0
M6	1250	19939.3	0.1	1.0	0.2	0.0

Supplementary Table S5

Higher-taxon composition of metazoan meiofaunal communities at stations around Misima. Data show mean (± SD) percentage total individuals (>250 µm) at each station, using corer drops as replicate samples.

Station	Depth (m)	Nematoda	Copepoda	Ostracoda	Others
M1	1380	28.9 ± 18.3	71.1 ± 18.3	-	-
M2	1461	24.1 ± 10.1	52.0 ± 5.5	22.7 ± 13.4	1.3 ± 2.3
M3	1467	59.7 ± 18.7	38.2 ± 18.8	2.2 ± 5.3	-
M4	1793	72.0 ± 2.7	23.0 ± 2.3	4.7 ± 1.7	0.3 ± 0.1
M5	1704	72.3 ± 8.2	23.0 ± 6.8	4.5 ± 2.1	0.2 ± 0.4
M6	1250	76.2 ± 2.7	21.4 ± 2.9	2.3 ± 0.3	0.1 ± 0.2

Supplementary Table S6

Higher-taxon composition of macrofaunal communities around Misima. Data show mean (± SD) percentage total individuals (>250 µm) at each station, using corer drops as replicate samples.

Station	Depth (m)	Polychaeta	Crustacea Peracarida	Mollusca	Echiura	Others
M1	1380	43.2 ± 3.1	0.5 ± 0.8	35.5 ± 7.8	18.4 ± 4.0	2.4 ± 1.7
M2	1461	72.8 ± 12.3	-	19.8 ± 10.3	1.1 ± 0.9	7.4 ± 2.0
M3	1467	71.5 ± 8.6	2.1 ± 2.0	18.7 ± 9.3	-	7.7 ± 3.7
M4	1793	68.4 ± 7.2	11.3 ± 1.7	16.1 ± 5.8	-	4.2 ± 1.0
M5	1704	63.2 ± 10.6	16.9 ± 5.8	14.8 ± 6.4	-	5.1 ± 1.8
M6	1250	54.2 ± 0.2	24.2 ± 1.1	16.9 ± 0.4	-	4.7 ± 0.7

Supplementary Table S7

Family-level composition of polychaete communities around Misima. Data show mean (± SD) abundance (ind. m^{-2}) at each station, using corer drops as replicate samples. The families listed are the five highest contributors to dissimilarity (SIMPER routine in Primer™ v.6) between the two station groups M1-M3 and M4-M6 recovered by cluster analysis (Fig. 6).

	Depth (m)	Spionidae	Paraonidae	Syllidae	Cirratulidae	Lumbrineridae
M1	1380	147 ± 74	199 ± 27	-	235 ± 74	211 ± 139
M2	1461	242 ± 101	62 ± 35	11 ± 19	212 ± 97	632 ± 449
M3	1467	286 ± 149	159 ± 126	18 ± 28	71 ± 68	492 ± 271
M4	1793	983 ± 174	867 ± 349	311 ± 244	474 ± 196	244 ± 112
M5	1704	393 ± 159	348 ± 72	122 ± 144	308 ± 87	37 ± 9
M6	1250	1337 ± 168	914 ± 149	893 ± 141	952 ± 220	75 ± 11

Supplementary Figure S1. Porewater concentrations of four ecotoxic trace metals in cores from four Misima stations. Data points for each depth interval are mean values from replicate cores (n = 3) at each station. Error bars are omitted for clarity. No measurements were made at stations M2 or M5.

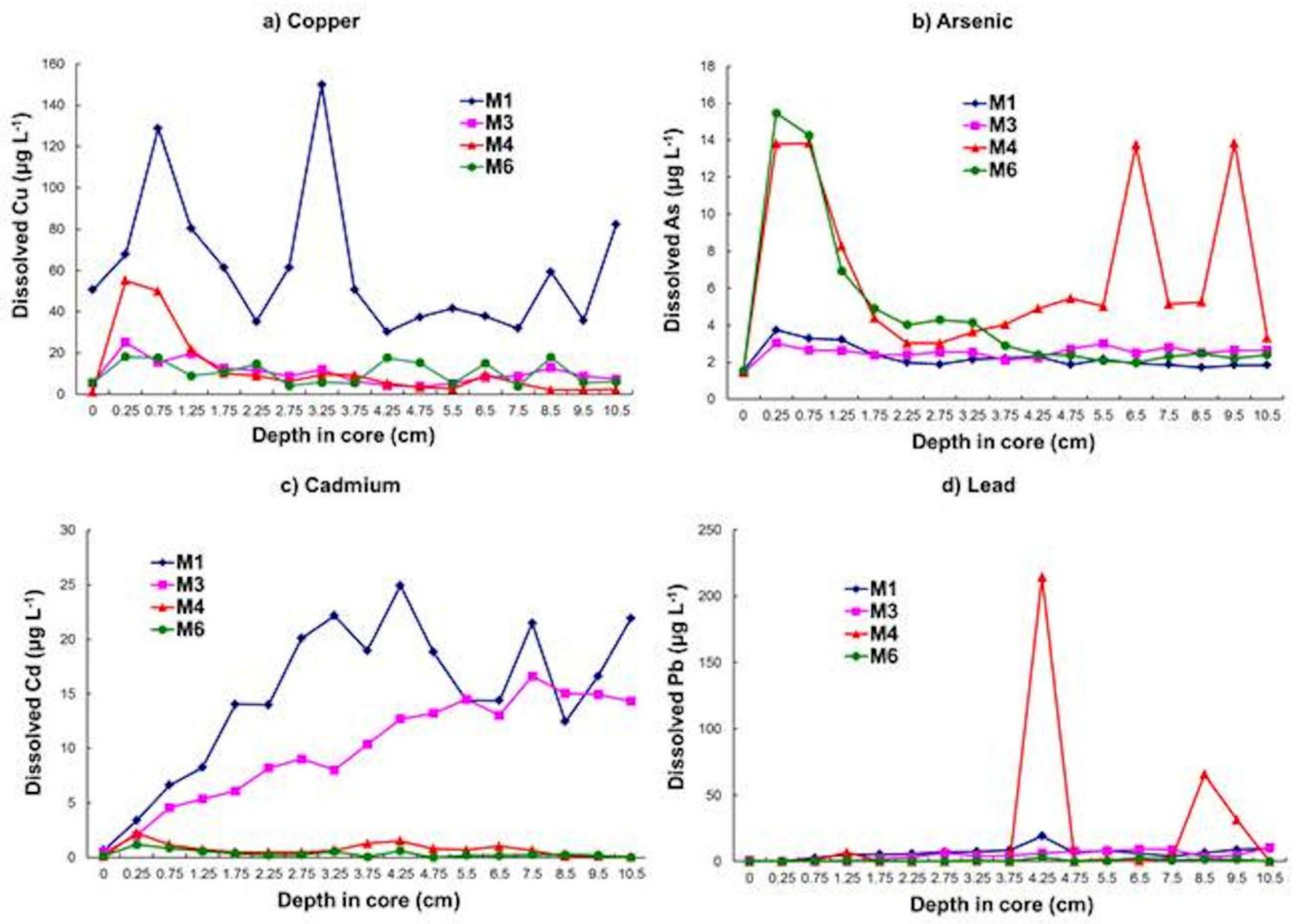

Supplement: Supporting Information — Supplementary Figures 1-3 and Supplementary Tables 1-3 [file srep09834-s1.pdf]
